# Enhanced extraction efficiency of natural D‐borneol from Mei Pian tree leaves pretreated with deep eutectic solvents

**DOI:** 10.1002/fsn3.1671

**Published:** 2020-06-02

**Authors:** Minghui Fu, Ziqing Lu, Xiaoyu Ma

**Affiliations:** ^1^ Bioengineering Department School of Biomedical and Pharmaceutical Sciences Guangdong University of Technology Guangzhou China

**Keywords:** deep eutectic solvent, extraction efficiency, Mei Pian tree, natural D‐borneol, pretreatment

## Abstract

Stems and leaves of Mei Pian tree can be used to extract natural D‐borneol. In this study, a variety of deep eutectic solvents (DESs) based on choline chloride were used to pretreat leaves of Mei Pian tree, from which natural D‐borneol was extracted by Soxhlet extraction. The extraction efficiency of most pretreated leaves was higher than that of the unpretreated leaves, and that pretreated by DES based on choline chloride and sucrose or choline chloride and glycol was maximal. Moreover, The Box–Behnken design (BBD) of response surface method was used to optimize the water content, temperature, and pretreatment time. The optimal conditions included pretreatment of Mei Pian tree for 4.07 hr at 44.5°C by DES, which was synthesized using choline chloride, sucrose, and water at the molar ratio of 5:2:5.9, and the extraction efficiency (4.936 mg/g) was maximal, more than twice as much as that of unpretreatment. The results showed that the pretreatment process using DES based on choline chloride and sucrose could effectively improve the extraction efficiency of natural D‐borneol from Mei Pian tree.

## Graphical abstract

Our study showed that the pretreatment process using deep eutectic solvent based on choline chloride and sucrose can effectively improve the extraction efficiency of natural D‐borneol from Mei Pian tree. This pretreatment method had potential in practical extraction of natural D‐borneol from Mei Pian tree.

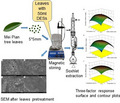

## 1 INTRODUCTION

A deep eutectic solvent (DES) is a mixture of two or more pure compounds for which the eutectic point temperature is below that of an ideal liquid mixture (Liu et al., 2018; Martins, Pinho, & Coutinho, 2019). In 2003, Abbott and his coworkers firstly used the term “DESs” to describe mixtures of amides with quaternary ammonium salts that had a much lower melting point than those of their pure compounds due to the formation of a hydrogen bonding complex. Next year, they synthesized DESs using choline chloride and carboxylic acids, and proposed DESs could be versatile alternatives to ionic liquids (Abbott, Boothby, Capper, Davies, & Rasheed, 2004). After that, DESs were deeply studied and applied in many fields. Because of their high boiling point, low melting point, renewability, biodegradability, low toxicity, and easy preparation, DESs hold great promise as solvents in many fields such as in the biomass treatment and biological separation, for example, hydrophilic DESs used in the extraction of phenolic acids, flavonoids, astaxanthin, anthocyanins, organic acids, stilbenes, alkaloids, Polysaccharides, vanillin, etc., (Li & Row, 2016; Rong et al., 2019) and hydrophobic DESs used in the extraction of polyprenyl acetates, artemisinin, and neonicotinoids (Cao et al., 2018; Cao, Yang, Cao, Wang, & Su, 2017; Krizek et al., 2018). Reportedly, DES based on choline chloride has good solubility for lignin and, hence, can be used frequently for the selective separation of lignin (Francisco, Bruinhorst, & Kroon, 2012; Francisco et al., 2013; Lynam, Kumar, & Wong, 2017; Malaeke, Housaindokht, Monhemi, & Izadyar, 2018; Kim, Dutta, Sun, Simmons, & Singh, 2018). Lignin and cellulose are the main components of the plant cell wall that can be dissolved by the DES treatment (Hou et al., 2018), so that the components inside the cell can be extracted easily (Kumar, Parikh, & Pravakar, 2016; Procentese et al., 2015). Therefore, DESs can be used in some pretreatment before extraction (Xu, Ding, Han, Dong, & Ni, 2016).

Mei Pian tree was a chemical type of *Cinnamomum camphora* discovered in the eastern part of the Guangdong province in China in 1980. Its leaves have abundant natural D‐borneol (C_10_H_18_O) which is a monoterpene and bicyclic organic compound with antibacterial (Shao‐Yun, Jian‐Yu, Shi, & Lin, 2012) and anti‐inflammatory (Juhas et al., 2008) activities. Also, Natural D‐borneol can protect the heart, brain, and other organs (Hur, Pak, Koo, & Jeon, 2013; Liu et al., 2011), regulate the central nervous system (Li et al., 2012; Zhang, Liu, & He, 2012), and promote the absorption of other drugs (Chen, Li, Su, & Chen, 2015; Chen et al., 2015; Chen et al., 2014). Thus, it has been widely used in the food and drug industries as an aromatic spice and a valuable medical material in southeast Asia (Chen et al., 2014). It can be synthesized by a chemical reaction or directly extracted from the plants (Zheng, 2011). The synthesized borneol may be mixed with the isoborneol and camphor so it might be toxic (Zeng & He 2004). Thus, the direct extraction of natural D‐borneol from plants was essential. So how to enhance the extraction efficiency is becoming more and more important. The conventional methods for extracting D‐borneol from the Mei Pian tree are water vapor distillation and organic solvent extraction. In both methods, D‐borneol is extracted directly from the leaves of Mei Pian tree without the pretreatment of leaves. After extraction, there is still considerable amount of D‐borneol confined inside the leaf cells due to the restriction of plant cell wall and cannot be extracted even with the highest extraction efficiency. In this study, we used a novel pretreatment method—pretreating the leaves of Mei Pian tree with DES—and found the extraction efficiency could be effectively improved after pretreatment.

## 2 MATERIALS AND METHODS

### 2.1 Materials

Mei Pian tree leaves and standard D‐borneol (99.0%) was provided by Guangdong Huaqingyuan Biological Science and Technology Co., Ltd. Choline chloride (99.7%), triethylene glycol (99.7%), urea (99.7%), and acetamide (99.7%) were purchased from Shanghai McLean Biochemistry Technology Co., Ltd. Glucose (99.7%), fructose (99.5%), maltose (99.5%), and sucrose (99.7%) were obtained from Chinese Medicine Group Chemical Reagent Co., Ltd. Other chemical reagents (99.7%) were procured from Tianjin Zhiyuan Chemical Reagent Co., Ltd.

### 2.2 Methods

#### 2.2.1 Extraction method

The leaves were cut by scissors into pieces about 5 × 5 mm size. The cut leaves were extracted with 60 ml ethanol and using the Soxhlet extraction apparatus in a constant temperature water bath at 95°C. The siphon occurred one time for about 1 hr until the color of the liquid in the Soxhlet extractor ceased to change. The extracted liquid in the flask was filtered and diluted with ethanol into 100 ml for testing.

#### 2.2.2 Determination of natural D‐borneol

The extraction was analyzed by GC‐MS with GCMS‐QP2010 plus (Shimadzu) equipped with a Rtz‐Wax column (30 m × 0.25 mm × 0.25 μm). The carrier gas helium was released at a flow rate of 1 ml/min. The inlet temperature was set at 220°C. A volume of 1µl samples (in ethanol) were injected manually in the splitless mode. The detector temperature was 250°C. The oven temperature was set from 70 to 100°C at 30°C/min and then to 250°C at 15°C/min, maintained for 2 min. An electron ionization system with ionization energy of 70 eV was used. The ion source temperature was set to 230°C, and the interface temperature was set to 250°C. The identification of natural D‐borneol was based on the comparison of the relative retention time with that of the standard samples at the same condition and by matching of the mass spectra of peaks with those obtained from standard spectra. Besides, the content of the natural D‐borneol was determined by comparing the area of peaks with the standard curve that was based on the different areas corresponding to different concentrations of standard samples. All tests were performed in triplicate. The extraction efficiency was calculated as follows: Extraction efficiency = The total extracted natural D‐borneol (mg)/ The total fresh sample (g).

#### 2.2.3 Preparation of DESs

All components used in the synthesis of DESs were vacuum‐dried at 80°C for 5 hr before using. The DESs were synthesized by mixing two components (hydrogen bond donor and hydrogen bond receptor) in a flat‐bottomed flask in a molar ratio listed in two columns on the left in Table 1. Subsequently, the mixture was heated at 80°C and agitated at 300 rpm for 2–3 hr to obtain a uniform and stable, transparent solution that was cooled to room temperature and stored in a vacuum drying oven.

**TABLE 1:** The extraction efficiency of D‐borneol from Mei Pian tree

Hydrogen bond donor	Choline chloride/Hydrogen bond donor(/water) (molar ratio)	Average of extraction efficiency (mg/g)±standard deviation
No	No pretreatment	2.011 ± 0.052^b^
triethylene glycol	1:4	2.546 ± 0.144^c^
glycerol	1:2	3.229 ± 0.068^gh^
glycol	1:2	3.514 ± 0.085^k^
oxalic acid	1:1	0.895 ± 0.074^a^
citric acid	1:1	3.081 ± 0.131^fg^
lactic acid	1:1	3.407 ± 0.126^hk^
urea	1:2	2.530 ± 0.133^c^
acetamide	1:2	2.938 ± 0.149^df^
glucose	5:2:5	2.211 ± 0.057^b^
fructose	5:2:5	2.843 ± 0.128^de^
maltose	5:2:5	3.345 ± 0.050^hk^
sucrose	5:2:5	3.561 ± 0.051^k^

Note

Different letters in the superscript indicate significant differences (*p* < .05). Ducan method in SPSS was used in statistical analysis.

#### 2.2.4 Pretreatment with DESs

An equivalent of 1 g cut leaves of Mei Pian tree were mixed with the 50 ml synthesized DES, stirred at the 300 rpm, and heated at 60°C for 5 hr. After pretreatment, the mixture was centrifuged at 663 *g* for 10 min. The cut leaves were placed in a sieve, then rinsed in running water for 2–10 min, wrapped in filter paper, and placed into Soxhlet apparatus to extract as described above. The extract was prepared to determine the content of D‐borneol by GC analysis, and the extraction efficiency was calculated.

#### 2.2.5 Single‐factor analysis of pretreatment condition

The effect of the different molar ratios of choline chloride to sucrose to water (2:5:2, 2:5:6, 2:5:10, 2:5:14, and 2:5:18), different temperatures (30, 45, 60, 75, and 90°C), and different pretreatment times (2, 4, 6, 8, and 10 hr) on the extraction efficiency was investigated. When one variable factor was studied, other factors were fixed. The pretreatment and extraction steps were conducted as described above.

#### 2.2.6 Optimization of the pretreatment condition using Box–Behnken design (BBD)

Based on the results of single‐factor experiments, the BBD of response surface methodology (RSM) (Design expert 8.0.6) was employed to optimize the three selected factors (water content (A, mol), pretreatment temperature (B, °C), and pretreatment time (C, h) for pretreatment of Mei Pian tree leaves that could enhance the extraction efficiency of natural D‐borneol. The three independent factors were investigated at three different levels (−1, 0, +1). The experimental design used in the current study was shown in Tables 2 and 3. The complete design consisted of 17‐run experiments.

**TABLE 2:** Factors and levels in BBD

Factor	Symbol	Level		
		−1	0	1
Water content (mol)	A	2	6	10
Temperature (°C)	B	30	45	60
Treatment time (h)	C	2	4	6

**TABLE 3:** The design and results of BBD

Run	A (mol)	B (°C)	C (h)	Extraction efficiency (mg/g)
1	0	1	−1	3.690
2	−1	0	1	2.862
3	0	1	1	3.711
4	0	−1	1	3.798
5	1	0	−1	2.596
6	0	0	0	5.158
7	−1	1	0	2.710
8	0	0	0	5.104
9	0	0	0	4.976
10	1	0	1	2.497
11	0	0	0	4.691
12	−1	0	−1	2.495
13	1	1	0	2.587
14	0	0	0	4.741
15	1	−1	0	2.619
16	0	−1	−1	3.734
17	−1	−1	0	2.831

Note

A represents water content, B represents pretreatment temperature, and C represents pretreatment time.

#### 2.2.7 Analysis by scanning electron microscope

Mei Pian tree leaves after no pretreatment, pretreatment with water, or pretreatment with DES of choline chloride and sucrose, or pretreatment with DES of choline chloride and glycol were dried at 55°C for 48 hr. The sample was attached to the sample table and coated with metal film, and then the surface structure of the sample was observed by scan electron microscopy of HITACHI UHR FE‐SEM SU8220 with the parameters including accelerated voltage 15.0 kV and UL probe. The effect of different pretreatments on the blade surface structure was analyzed.

## 3 RESULTS AND DISCUSSION

### 3.1 Extraction efficiency of different pretreatments

After GC‐MS detection, it was found that the retention time of the highest content in the extract was consistent with that of the standard D‐borneol, and its mass spectrum was also highly consistent with that of D‐borneol (up to 97% identity) (Figure S1and S2). This indicated the main component of the extract was D‐borneol, and it was consistent with our previous report (Chen, Su, Li, Li, & Li, 2011). According to the different areas corresponding to different concentrations of standard D‐borneol, we depicted the standard curve. The effective concentration range was 0.5–3 ug/mL, and the correlation coefficient was 0.99. The following contents of D‐borneol in the extract were determined according to this standard curve.

The extraction efficiencies of Soxhlet's extractions of natural D‐borneol from the Mei Pian tree that were not pretreated or pretreated using various DESs were shown in Table 1. Twelve types of DESs were synthesized as the hydrogen bond receptor using choline chloride. The hydrogen bond donors included organic acids, polyols, amides, and sugars. Table 1 showed that all extraction efficiencies with DES pretreatment were higher than those without pretreatment except for those pretreated with DES synthesized using glucose or oxalic acid. On the other hand, the extraction efficiencies of leaves pretreated with glycol‐based DES and sucrose‐based DES were significantly higher than those pretreated with other DESs except for maltose‐based DES and lactic acid‐based DES that did not differ significantly from glycol‐based DES and sucrose‐based DES. Therefore, we selected the cheaper and effective DES prepared with sucrose and choline chloride for the pretreatment of Mei Pian tree leaves prior to extraction in the subsequent optimization of the pretreatment condition. The hydrogen donors and receptors in DESs could interact with lignin or cellulose in the cell wall and facilitate the loosening of lignin and cellulose structures in the cell structures. Therefore, after pretreatment with DES, the cell wall structure was loosened, and the intracellular substances were extracted easily, and hence, the maximal extraction efficiency was higher than that without pretreatment, which was consistent with that reported previously (Lu et al., 2016). The current results also showed when choline chloride was used as the hydrogen bond receptor, the pretreatment efficiency varied with the types of hydrogen bond donors. For the polyol‐based DES pretreatment, the extraction efficiencies were decreased with the increase of hydrophilicity of the hydrogen bond donors. The more hydroxyl groups the hydrogen bond donors contained, the lower the extraction efficiencies, which was consistent with the study by Hou et al. (2018) and Dai et al. (2013). Both studies demonstrated that excessive hydroxyl groups in the hydrogen bond donors might lead to extensive hydrogen bond structure formation, thereby providing stable DESs with higher viscosity. Hence, additional energy was required to loosen the bonded structures to enable interactions with the solute. Thus, in our results, the order of pretreatment efficiency was triethylene glycol < glycerol < glycol in the polyol‐based DESs because of the order of the hydrogen bond strength of the three polyol‐based DESs was triethylene glycol > glycerol > glycol. Similarly, the pretreatment efficiency of amide‐based DESs altered as the hydrogen bond donor structures changed. DES with acetamide as the hydrogen bond donor had higher extraction efficiency than DES with urea as the hydrogen bond donor. The pretreatment effect of DESs formed by sugar was not directly related to the number of hydroxyl groups because of the addition of water in our study. Water could also be involved in the formation of hydrogen bonds and affect the viscosity of DESs. A majority of the sugar DESs had high viscosity, which was not conducive to sufficient contact between the leaves of Mei Pian tree. The addition of water to the system could reduce the viscosity, and the contact between the DESs and the leaves of Mei Pian tree increased. Consequently, not viscosity, but the interaction between the DESs and the extract primarily influenced the extraction efficiency. DESs synthesized by disaccharides such as maltose and sucrose had stronger interaction than those synthesized by monosaccharides such as glucose and fructose. Thus, disaccharide‐DES exerted a superior effect on the extraction of pretreatment. In the current study, in the organic acids‐based DESs, the stronger the acidity, the worse the pretreatment effect. Since lignin is an alkaline‐soluble biopolymer (Adler, 1977), organic acid‐based DESs with alkalescence could facilitate lignin solubilization and improve extraction efficiency. The extraction efficiency of the DES based on oxalic acid with the strongest acidic was the lowest, even lower than that of the nonpretreated solvent. It can be speculated that oxalic acid might combine with the components of the cell wall to form a stronger structure that might resist the release of natural D‐borneol.

Although increasing the yield of D‐borneol by DES pretreatment would result in additional production costs, the recycling and reuse of the DES utilized in the pretreatment will be beneficial to the large‐scale industrial production with high economic benefits.

### 3.2 Optimization of pretreatment conditions

The pretreatment conditions of DES prepared with sucrose, choline chloride, and water were optimized from three aspects: water content, temperature, and pretreatment time (Figure S3‐S5).

Figure S3 showed that the extraction efficiency of natural D‐borneol increased when the water content increased from 2 to 6 mol but decreased when from 6 to 10 mol. Further increasing the water content resulted in the slight fluctuation of the extraction efficiency of D‐borneol; however, the value was lower than the extraction efficiency of that with 6 mol water content. Therefore, the optimization of the water content was 6 mol.

The DES synthesized by choline chloride and sucrose had a high viscosity with little water, during the pretreatment, which was not conducive to the thorough contact between the solvent and the material. However, when the water content is excessive, the extraction efficiency of D‐borneol showed a downward trend, because, with the increase in the water content, the contact probability of the extracts (components of the cell wall) and water molecules increased during pretreatment, while the contact probability with solvent decreased, which affected the pretreatment effect.

With an increase in the temperature, the extraction of D‐borneol firstly showed an increasing trend (Figure S4), due to the high viscosity of DES at room temperature, which rendered difficulty in thoroughly soaking the extract. However, when the temperature exceeded 45°C, the extraction efficiency began to decrease, which might be caused by the loss of natural D‐borneol due to the high temperature.

When the pretreatment time was prolonged from 2 to 4 hr, the extraction efficiency of D‐borneol was maximal (Figure S5), which indicated that 2 hr was not sufficient for the complete pretreatment of Mei Pian tree leaves. When the pretreatment time was >4 hr, the extraction efficiency of D‐borneol decreased slowly, which indicated that the effect of pretreatment was not improved by extending the pretreatment time; this might even result in the loss of D‐borneol.

According to the results of the above single‐factor experiment, BBD analysis was completed with three factors, including water content, temperature, and pretreatment time. The three levels of water content were 2, 6, and 10 mol. The three levels of pretreatment temperature were 30, 45, and 60°C. The three levels of pretreatment time were 2, 4, and 6 hr. According to the Design‐Expert test design (Table 2), the results were illustrated in Table 3.

Design‐Expert was used to simulate the results of BBD. The quadratic polynomial regression equations of extraction efficiency Y versus water content A, treatment temperature B, and treatment time C were obtained as the following:

Y=−0.075A‐0.035B+0.044C+0.022AB−0.12AC−0.011BC‐1.68A^2^−0.56B^2^−0.64C^2^+4.93

The variance analysis of the above equations was shown in Table 4. According to the analysis of variance, *p*‐value (<.0001) of three‐factor global model was significant. The model *F*‐value of 69.75 implied that the model was significant. However, there was only a 0.01% chance that an *F*‐value this large could occur due to noise. The quadratic coefficients (A^2^, B^2^, or C^2^) also had significant effect, while the linear coefficients (A, B, and C) and the interaction between the two factors of the water content, pretreatment temperature, and pretreatment time had insignificant effect. The R^2^_Pred_ (0.9785) was in reasonable agreement with the R^2^_Adj_ (0.9748) with < 0.02. As the lack of fit was not significant (*p*‐value = .9892), this proposed model could predict the extraction efficiency according to the parameters of water content, pretreatment temperature, and pretreatment time. The correlation coefficient *R* ^2^ = .9890 > .9. Therefore, the above model can be employed to predict the pretreatment effect of DES on the leaves of Mei Pian tree.

**TABLE 4:** Analysis of variance of BBD

Source	Sum of squares	*df*	Mean square	*F* value	*p*‐value
Model	16.32	9	1.81	69.75	<.0001*
A‐A	0.045	1	0.045	1.72	.2305
B‐B	0.010	1	0.010	0.39	.5532
C‐C	0.016	1	0.016	0.60	.4643
AB	1.980E−003	1	1.980E−003	0.076	.7905
AC	0.054	1	0.054	2.09	.1917
BC	4.623E−004	1	4.623E−004	0.018	.8977
A^2^	11.94	1	11.94	459.21	<.0001*
B^2^	1.34	1	1.34	51.37	.0002*
C^2^	1.71	1	1.71	65.81	<.0001*
Residual	0.18	7	0.026		
Lack of Fit	4.877E−003	3	1.626E−003	0.037	.9892
Pure Error	0.18	4	0.044		
Cor Total	16.51	16			

Note

* is a mark that *p*‐value < .01.

**FIGURE 1:**
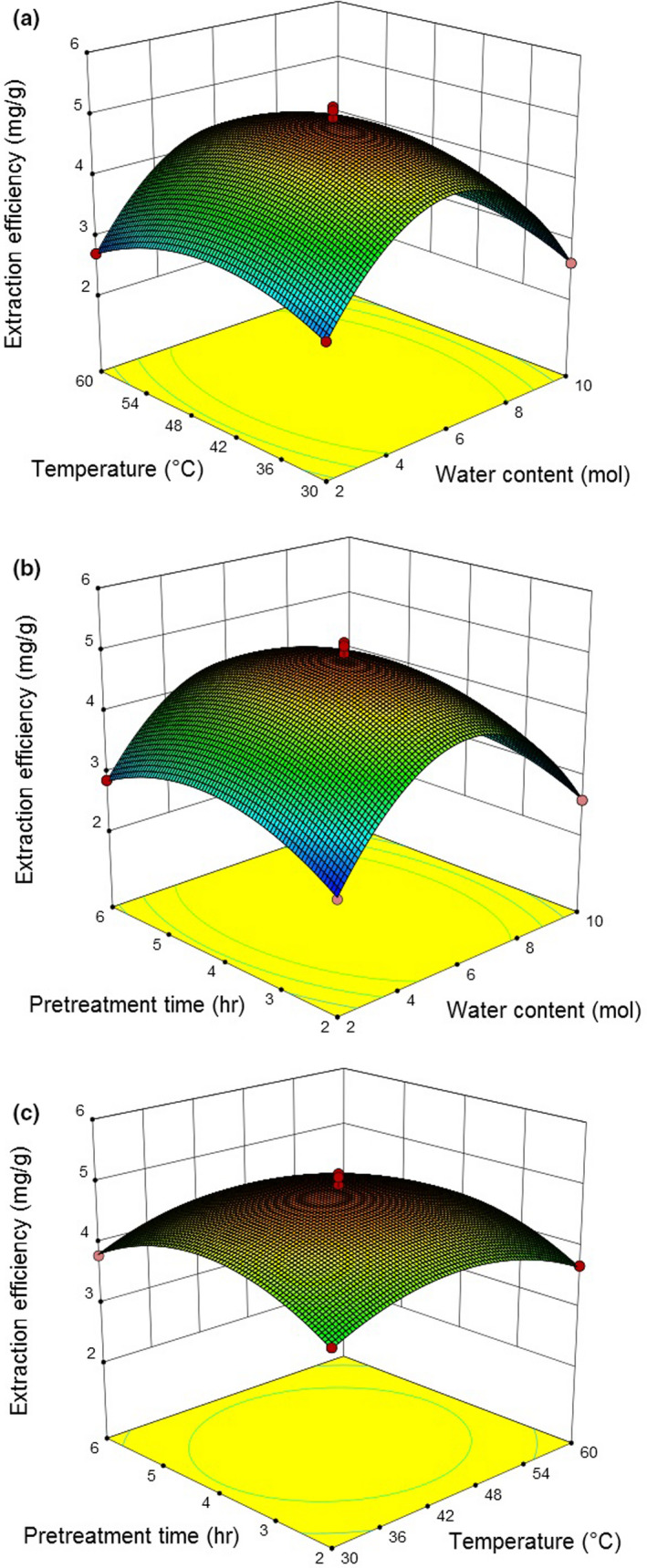
Response surface and corresponding contour plots of three factors on the extraction of natural D‐borneol. (a) The effect of water content and temperature on extraction efficiency when the pretreatment time was set at 4 hr; (b) The effect of water content and pretreatment time on extraction efficiency when the temperature was 45°C; (c) The effect of temperature and pretreatment on extraction efficiency when the water content was 6 ml

The contour diagram was illustrated in Figure 1. The optimum conditions were determined by the regression equation: Molar ratio of water content was 5:2:5.9, pretreatment temperature 44.5°C, treatment time 4.07 hr. Under these conditions, the extraction efficiency of natural D‐borneol was 4.936 mg/g.

The three‐dimensional response surface plots were employed to visualize the correlations between dependent and independent variables, in which one constant variable at its intermediate level and two different variables within their test range were revealed (Zhang, Liu, Li, & Chi, 2014). As shown in Figure 1a, the extraction efficiency increased first, followed by a decrease with the increasing temperature and water content when the pretreatment time was 4 hr. The extraction efficiency was maximum at 4.9354 mg/g at the water content of 5.91 mol and at the temperature of 44.51°C. When the temperature was set at 45°C, the extraction efficiency initially increased and then decreased slowly (Figure 1b), from 2 to 10 mol or as the pretreatment time increased from 2–6 hr, among which the maximum extraction efficiency was observed at 4.9357 mg/g when the water content was 5.91 mol and the pretreatment time was 4.07 hr. For the fixed water content of 6 mol, the extraction efficiency significantly improved as the temperature increased from 30–44.51°C and the pretreatment time increased from 2 hr to 4.07 hr; the maximum efficiency was 4.9353 mg/g, followed by a decrease (Figure 1c). Therefore, the predicted maximum extraction efficiency (4.936 mg/g) was obtained under the estimated optimal conditions (water content was 5:2:5.9, pretreatment temperature was 44.5°C, and treatment time was 4.07 hr), which could be calculated by the regression equation.

### 3.3 Electron microscope analysis

**FIGURE 2:**
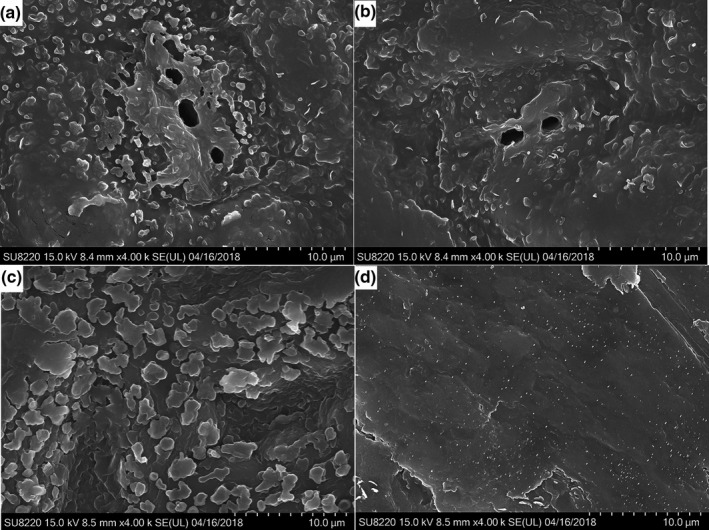
Configuration of surface of leaves pretreated by different methods. (a) The configuration of surface of leaves pretreated by DES synthesized by choline chloride and glycol; (b) The configuration of surface of leaves pretreated by DES synthesized by choline chloride and sucrose; (c) The configuration of surface of leaves pretreated by water; (d)The configuration of surface of leaves untreated; magnification = 104

The surface structure of the leaves was observed by field emission scanning electron microscope (SEM) after drying the leaves in water treatment, unpretreated, or pretreated with DES synthesized by choline chloride and glycol or DES synthesized by choline chloride and sucrose (Figure 2). The leaf surface of Mei Pian tree was smooth and compact, while the surface of leaves treated by water and by DES treatment was rough, and after DES treatment, several pores appeared on the surface of the leaves. The porous morphology could be attributed to the solubility of the DES to lignin and other components of the plant cell wall, thereby changing the overall structure of the leaf surface. The porous structure allowed the material across the cell and improved extraction efficiency.

## 4 CONCLUSIONS

Pretreatment by most DESs based on choline chloride could effectively improve the extraction efficiency of natural D‐borneol from Mei Pian tree. Among them, the extraction efficiency of pretreatment by DES based on choline chloride and sucrose or choline chloride and glycol was maximal. The pretreatment conditions optimized by BBD method included pretreatment of Mei Pian tree for 4.07 hr at 44.5°C by DES, which was synthesized using choline chloride, sucrose, and water at the molar ratio of 5:2:5.9, and the extraction efficiency (4.936 mg/g) arrived at maximum, more than twice as high as that of unpretreatment.

## CONFLICT OF INTEREST

The authors declare that they have no conflict of interest.

## ETHICAL STATEMENT

Ethics approval was not required for this research.

## Supporting information

Supinfo
