# Supplementary material for: Enhanced extraction efficiency of natural D‐borneol from Mei Pian tree leaves pretreated with deep eutectic solvents
Source: Food Sci Nutr. 2020 Jun 2;8(7):3806–13. doi: 10.1002/fsn3.1671 (PMC7382189; doi:10.1002/fsn3.1671)
Supplement: Supplementary file 1 — Supinfo [file FSN3-8-3806-s001.doc]

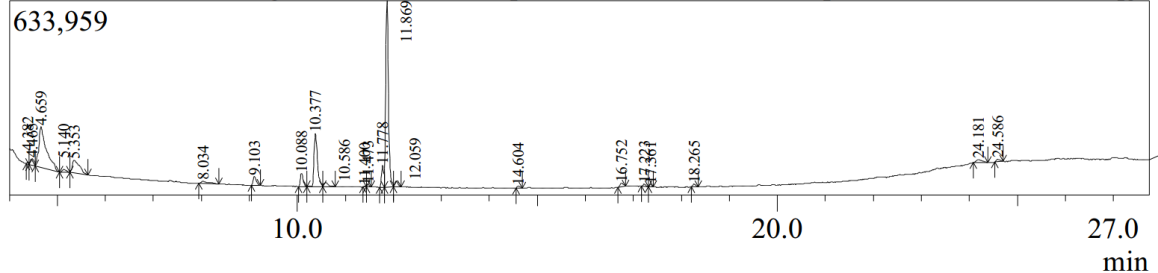


**FIGURE 1**  Gas chromatogram of the extraction


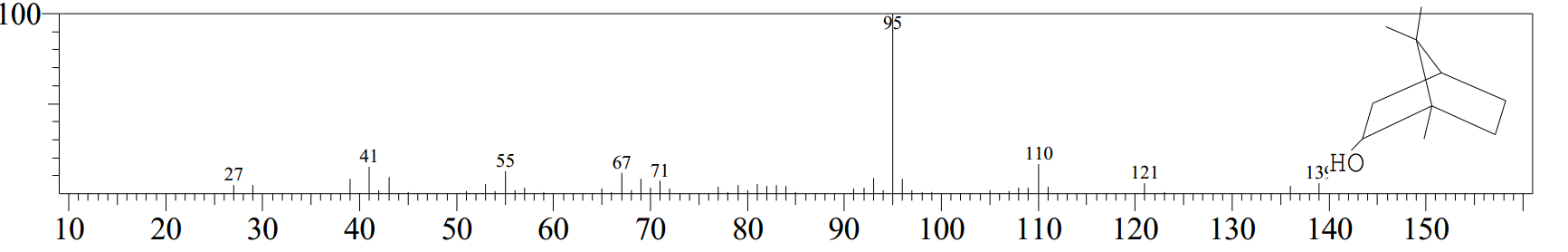


A


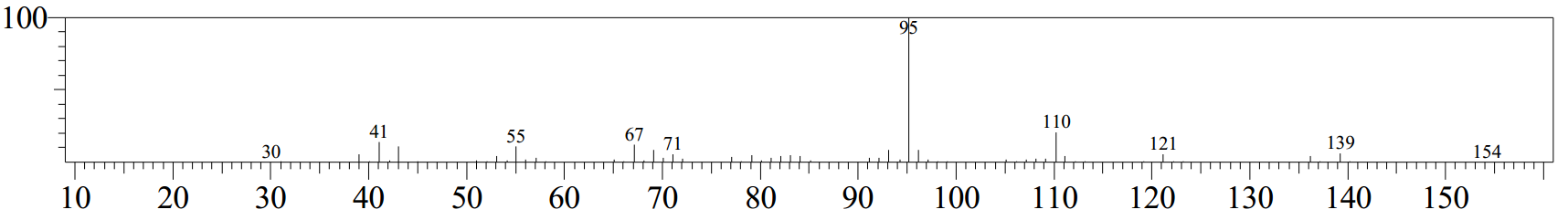


B

**FIGURE 2**  Mass Spectrometry of D-borneol (A) and the highest peak in the gas chromatogram (B)


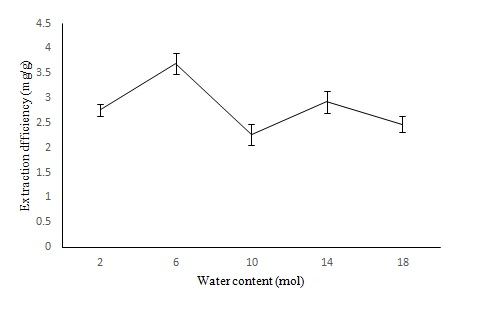


**FIGURE 3**  Effect of water content in DES on extracion efficiency.

**
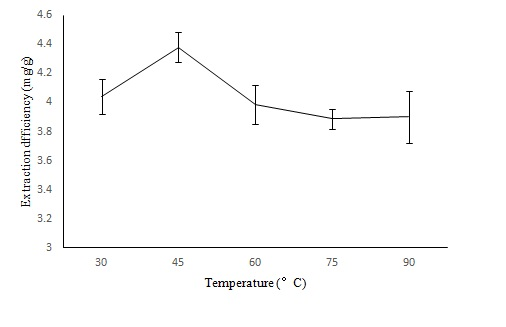
**

**FIGURE 4** Effects of pretreatment temperature on extraction efficiency.

**
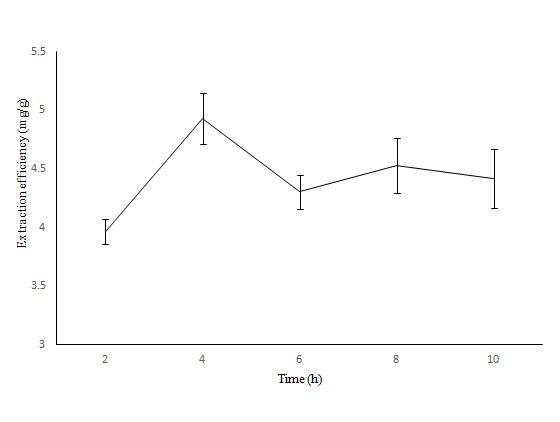
**

**FIGURE 5**  Effect of treatment time on extraction efficiency.
